# Early Switch to Fulvestrant Plus Palbociclib Improves Outcomes in *ESR1*-Mutated, Estrogen Receptor-Positive Metastatic Breast Cancer

**DOI:** 10.1093/oncolo/oyac016

**Published:** 2022-03-28

**Authors:** Anne Jacobson

## Abstract

For patients with estrogen receptor-positive metastatic breast cancer who are treated with palbociclib plus an aromatase inhibitor, the strategy of switching to palbociclib plus fulvestrant upon detection of a resistance mutation more than doubled progression-free survival, according to results from the phase III PADA-1 trial.

Estrogen receptor gene (*ESR1*) mutations restore the ligand-independent activity of the estrogen receptor (ER) in patients with ER-positive metastatic breast cancer. Although these mutations are associated with resistance to aromatase inhibitor (AI) therapy, tumors harboring mutated *ESR1* retain their sensitivity to selective estrogen receptor degraders (SERDs). Mutations in *ESR1* are rare at diagnosis, occurring in <5% of patients with metastatic breast cancer. However, the prevalence of *ESR1* mutations increases to 30% to 40% among patients whose disease progresses on first-line aromatase inhibitor therapy.

The presence of *ESR1* mutations in the blood (*bESR1*), detected by cell-free circulating DNA analysis, is associated with resistance to aromatase inhibitor therapy, but not to treatment with fulvestrant or palbociclib.

The phase III PADA-1 trial was designed to evaluate the feasibility of preventing or delaying tumor progression in patients receiving first-line treatment with palbociclib plus aromatase inhibitor therapy by switching from an AI to fulvestrant as soon as a *bESR1* mutation becomes detectable, while also maintaining treatment with palbociclib.

François-Clément Bidard, M.D., Ph.D., of the Institut Curie and Paris-Saclay University, presented findings from the PADA-1 trial.^1^

## Study Design

The PADA-1 trial enrolled 1,017 patients with ER-positive/HER2-negative metastatic breast cancer who were undergoing first-line treatment with palbociclib plus an aromatase inhibitor. Patients provided blood samples for *bESR1* mutation screening at enrollment, at month 1, and every 2 months thereafter. Blood samples were monitored for several *ESR1* mutations: E380, P535, L536, Y537, and D538.

Monitoring revealed a new *ESR1* mutation in 172 patients who did not experience concurrent disease progression. The median time from trial enrollment to detection of the *ESR1* mutation was 14.2 months (range, 2.8 to 47.1 months). Patients with a newly detected mutation were randomly assigned to 1 of 2 treatment approaches:

- Maintain treatment with palbociclib plus an aromatase inhibitor (*n* = 84)
- Switch treatment to palbociclib plus fulvestrant (*n* = 88)

The co-primary endpoints were progression-free survival (PFS) and grade ≥3 hematologic adverse events.

Baseline characteristics were similar in both treatment groups (Table 1). The median patient age was approximately 61 years, and one-third of patients (34%-37%) had prior treatment with an aromatase inhibitor.

**Table 1.** Baseline characteristics in ER-positive, *ESR1*-mutated metastatic breast cancer

Characteristics	Palbociclib plus aromatase inhibitor (*n* = 84)	Palbociclib plus fulvestrant (*n* = 88)
Median age	60 years	62 years
Prior adjuvant aromatase inhibitor therapy		
Yes	37%	34%
No	63%	66%
Metastatic sites		
Bone only	23%	22%
Visceral	49%	48%
Non-visceral ± bone	29%	31%
ECOG performance status		
0	61%	57%
1-2	39%	43%

## Key Findings

After a median follow-up of 26 months, the strategy of switching patients from an aromatase inhibitor to fulvestrant upon *bESR1*-mutation detection was associated with a 39% reduction in the risk of disease progression or death (Table 2). The median PFS for patients who were switched to fulvestrant was 11.9 months, compared with 5.7 months for patients who remained on an aromatase inhibitor (HR, 0.61; *p* =.005).

**Table 2.** Progression-free survival in ER-positive, *ESR1*-mutated metastatic breast cancer

Endpoint	Palbociclib plus aromatase inhibitor (*n* = 84)	Palbociclib plus fulvestrant (*n* = 88)	Stratified HR(95% CI)	*p* value
Median PFS	5.7 months	11.9 months	0.61(0.43-0.86)	.005

Among those who were randomized to the aromatase inhibitor arm, 69 patients progressed and were given the option to crossover to the fulvestrant arm. Of those who switched to fulvestrant (*n* = 47), the second median PFS was 3.5 months. This suggests that second-line fulvestrant confers a benefit for a brief duration and underscores the importance of detecting *ESR1* mutations early.

The analysis of the co-primary endpoint of grade ≥3 hematologic adverse events found no safety signals associated with switching from an aromatase inhibitor to fulvestrant (Table 3). Dose reductions were similar in the palbociclib plus aromatase inhibitor (7.1%) and palbociclib plus fulvestrant (7.9%) groups. One patient in the fulvestrant group (1.1%) withdrew from the trial due to a treatment-related adverse event.

**Table 3.** Grade 3-4 adverse events in patients with ER-positive metastatic breast cancer

Adverse event	Palbociclib plus AI (*n* = 84)	Palbociclib plus fulvestrant (*n* = 88)
Leukopenia		
Grade 3	6.0%	6.8%
Neutropenia		
Grade 3	34.5%	36.4%
Grade 4	2.4%	0%
Anemia		
Grade 3	2.4%	0%
Thrombocytopenia		
Grade 3	1.2%	2.3%
Nausea		
Grade 3	1.2%	0%
Pneumopathy		
Grade 3	3.6%	0%
Pain		
Grade 3	1.2%	4.5%
Other		
Grade 3	6.0%	0%
Grade 4	0%	1.1%

Findings from the PADA-1 trial support a personalized approach to treatment modification based on the early detection of *ESR1* mutations in patients with ER-positive metastatic breast cancer. Results also demonstrate the utility of targeting the brief window of opportunity—after the initiation of first-line therapy but before tumor progression—to improve patient outcomes in patients who develop resistance mutations.

## Notes

Please note that this content was created for the purpose of summarizing the content of the San Antonio Breast Cancer Symposium 2021 and has not been peer reviewed under the standard policies of *The Oncologist*.
